# Enhanced DMA Test Procedure to Measure Viscoelastic Properties of Epoxy-Based Molding Compound: Multiple Oscillatory Strain Amplitudes and Monotonic Loading

**DOI:** 10.3390/mi16040384

**Published:** 2025-03-27

**Authors:** Sukrut Prashant Phansalkar, Roshith Mittakolu, Bongtae Han, Taehwa Kim

**Affiliations:** 1 Department of Mechanical Engineering, University of Maryland, College Park, MD 20742, USA; sukrutp@umd.edu (S.P.P.); mroshith@umd.edu (R.M.); 2 Hyundai Motors R&D Division, Hwaseong-si 18280, Gyeonggi-do, Republic of Korea; thkim1@hyundai.com

**Keywords:** dynamic mechanical analysis (DMA), epoxy molding compounds (EMC), frequency domain analysis, time domain analysis

## Abstract

Dynamic mechanical analysis (DMA) is routinely practiced in the semiconductor industry to measure the viscoelastic properties of various thermosetting polymers. Modern commercial DMA test machines are highly-advanced systems which enable users to perform automatic testing and post-processing of the experimental data. When highly filled thermosets like epoxy-based molding compound (EMC) are tested, unique challenges are encountered during measurements due to the extremely large change in modulus over the testing temperature range. An advanced procedure is proposed to cope with these problems. The first part is the use of different oscillation strain amplitudes so that the variations in stress amplitudes across the testing domain remain consistent. The second part is the conducting of two monotonic tests at the lowest and highest temperatures to obtain the glassy modulus and equilibrium modulus, which can guide the master curve construction accurately. The results of the proposed procedure are presented. The relaxation modulus master curve is used to conduct a virtual testing to verify the accuracy of the generalized Maxwell model constants determined from the frequency data using the proposed procedure.

## 1. Introduction

Dynamic Mechanical Analyzer (DMA) has become a tool that almost every semiconductor packaging industry routinely employs to measure the viscoelastic properties of thermosetting polymers used in the packaging technologies [1,2,3,4,5]. The procedure involves the application of sinusoidal displacement (strain) of a known frequency to a specimen and the measurement of a force (stress) response. The phase lag between stress and strain response provides insights into various aspects of viscoelasticity [6,7,8].

The theory (i.e., dynamic mechanical analysis) is very mature, and modern commercial DMA machines provide versatile operational capabilities, through which users can readily select various loading options such as force track, automatic temperature ramping, soaking, etc. They also come with an advanced user interface (UI) S/W, which performs automatic post-experiment data analyses such as time–temperature superposition (TTS), the identification of glass transition temperature ($T_{g}$), etc. This level of turnkey automation allows a *set-and-forget* style of operation, which has reduced human labor/effort and measurement uncertainties significantly during routine practice.

One of the most important thermoset-based materials used in semiconductor packaging are epoxy-based molding compounds (EMCs). They are used to encapsulate semiconductor chips and other internal circuitry of electronic packages through molding processes. The characterization of the mechanical properties of EMCs has become more important as EMCs have become a part of advanced interconnect systems in various heterogeneous integration solutions, including fan-out wafer-level packaging (FO-WLP) and embedded technologies, where vias and signal redistribution layers are fabricated in or on EMCs after molding is completed [9].

**Figure 1:**
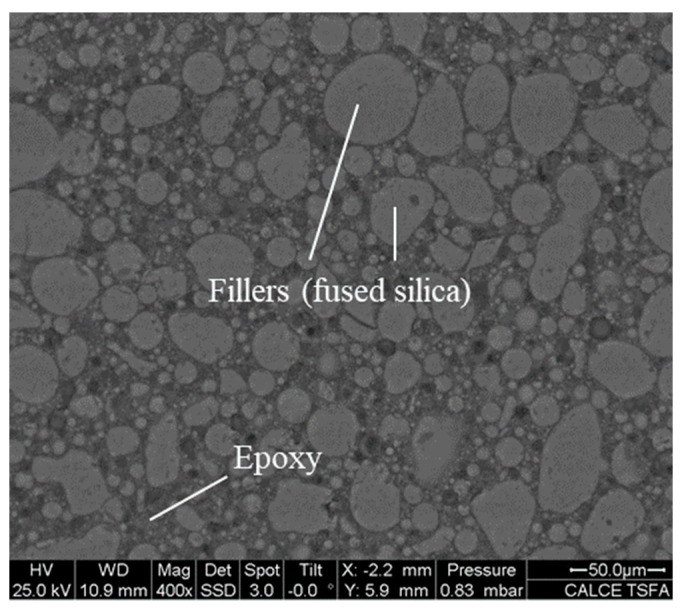
SEM image of epoxy-based molding compound (EMC).

EMCs are composite materials containing very high contents of silica filler (80 to 90% wt./wt.). A scanning electron microscope (SEM) image of a typical EMC is shown in Figure 1. EMCs undergo a significant change (≈one to two orders of magnitude) in modulus over the thermal excursion encountered in the manufacturing processes of semiconductor packages. This large change in modulus poses technical challenges against the *set-and-forget style* of operation. In fact, blind operation can produce significant errors in viscoelastic property measurements. Although the theory behind DMA is solid, its practice should be reviewed for advanced EMCs, and this is the motivation of this paper.

This paper first reviews the well-known theory behind the relaxation of a viscoelastic polymer in the time and frequency domains for the sake of readers’ convenience. This is followed by describing how DMA has been practiced for the routine measurement of time-dependent viscoelastic properties, namely by using a single set of input parameters. The implementation challenges associated with highly filled thermosets, i.e., EMCs, are discussed. Finally, an improved testing procedure is proposed in order to cope with the challenges. The improved procedure is implemented to measure the viscoelastic properties of an EMC over a large temperature range. Finally, determination of the Prony series and time domain master curve is discussed. The improvement is corroborated by conducting virtual DMA testing.

## 2. Relationship Between Time Domain Analysis and Frequency Domain Analysis

The well-known theories behind time domain linear viscoelastic (LVE) analysis and dynamic mechanical analysis (DMA) are reviewed briefly.

### 2.1. Time Domain Analysis for Thermorheologically Simple (TRS) Materials

**Figure 2:**
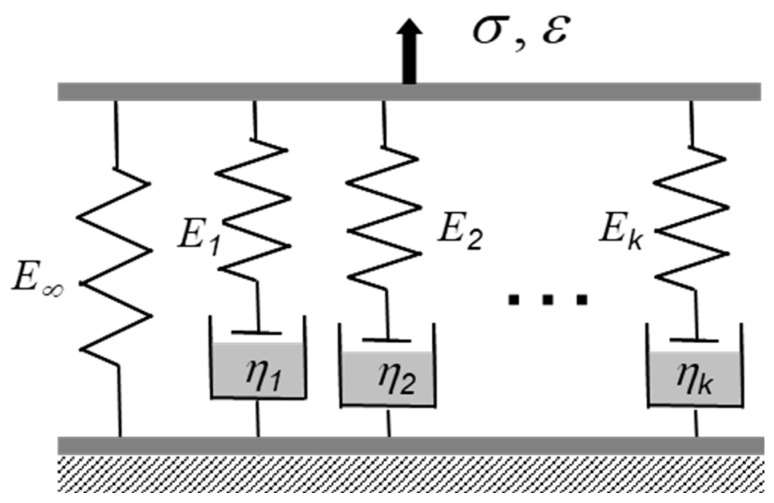
Generalized Maxwell model.

For a general polymer exhibiting linear viscoelasticity (LVE), the viscoelastic behavior under a stress relaxation testing condition can be expressed by the generalized Maxwell model, which consists of N viscoelastic chains [7]. A schematic diagram of the generalized Maxwell model is shown in Figure 2. Based on the model, Young’s (or the uniaxial) relaxation modulus, E(t), can be expressed using a Prony series:

(1) $$E\left(t\right)=E_{\infty }+\sum_{i=1}^{N}E_{i}\exp\left(-\frac{t}{\tau_{i}}\right)\;$$

where $E_{i}$ and $\tau_{i}$ are the modulus (or stiffness) and the relaxation time constants, respectively, and $E_{\infty }$ is the equilibrium modulus (the modulus of the fully relaxed polymer). The glassy modulus ($E_{G}$) is defined as the point where no relaxation has occurred. Mathematically, it is defined as follows:

(2) $$E_{G}=E_{\infty }+\sum_{i=1}^{N}E_{i}$$

The relationship between time-dependent stress, $\sigma \left(t\right),$ and applied strain, ε(t), can be expressed by the following hereditary integral:

(3) $$\sigma \left(t\right)=\int_{-\infty }^{t}E\left(t-\tau \right)\frac{\partial \varepsilon }{\partial \tau }d\tau$$

where the stress response can be determined by evaluating the integral over the history of strain loading [7].

**Figure 3:**
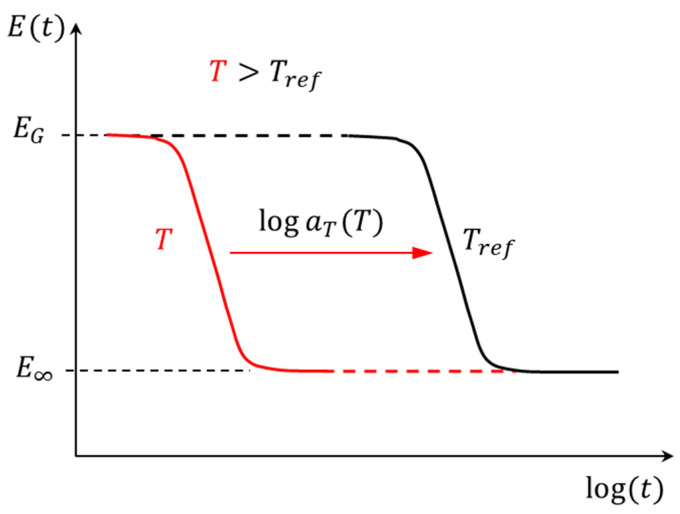
Illustration of material behavior of TRS polymers in time domain.

It is well known that, for thermorheologically simple (TRS) polymers, their viscoelastic behavior at one temperature can be related to that at another temperature only by a change in the log time scale. The TRS fundamental is illustrated schematically in Figure 3, where the relaxation modulus at any temperature, T, is determined by shifting the master curve (at $T_{ref})$ on a log time scale using a temperature-dependent shift factor, $a_{T}$. At any temperature $T>T_{ref}$, the relaxation rate is faster (shifted to the left of $T_{ref})$, while for $T<T_{ref}$, the relaxation rate is slower (shifted to the right of $T_{ref}).$

This behavior is known as the time–temperature superposition (TTS). The relaxation modulus at any temperature, T, can be expressed as

(4) $$E\left(t,T\right)=E_{\infty }+\sum_{i=1}^{N}E_{i}\exp\left(-\frac{\xi \left(T\right)}{\tau_{i}}\right)$$

where $\xi \left(T\right)$ is the reduced time, which is defined as follows:

(5) $$\xi \left(T\right)=\frac{t}{a_{T}(T)}$$

**Figure 4:**
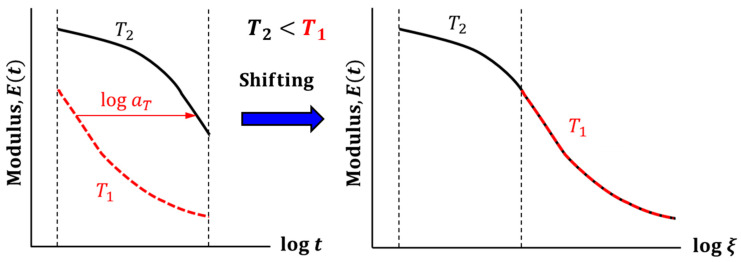
Schematic illustration of TTS shifting using short segments of master curve.

In practice, it is impractical to measure the master curve in a single experiment due to the large time range involved in the master curve. Instead, the relaxation tests are conducted at various temperatures to produce short segments of the master curve. These short segments are shifted along the log time axis to create a complete relaxation master curve. This is illustrated schematically in Figure 4. Two segments, at temperature $T_{1}$ and $T_{2}$ ($T_{1}>T_{2}$), obtained from a stress relaxation test are shown. The segment at $T_{1}$ is shifted on the log time scale until it overlaps with $T_{2}$ to create one continuous segment. The amount of shifting is the shiftfactor, $a_{T}$.

### 2.2. Frequency DomainAnalysis for TRS Materials

Dynamic mechanical analysis (DMA) involves measuring the stress–strain relationship while applying a cyclic strain, $\varepsilon \left(t\right)$, at a known frequency, ω. For viscoelastic materials, a phase lag, δ, exists between the applied cyclic strain and the measured cyclic stress, $\sigma \left(t\right).$

The ratio of cyclic stress to strain is called dynamic (or complex modulus), $\hat{E}\left(\omega \right)$, which is defined as follows:

(6) $$\varepsilon \left(t\right)=\varepsilon_{a}\sin\left(\omega t\right)=\varepsilon_{a}e^{j\omega t}\;\sigma \left(t\right)=\sigma_{a}\sin\left(\omega t+\delta (\omega )\right)=\sigma_{a}e^{j\left(\omega t+\delta (\omega )\right)}\;\frac{\sigma \left(t\right)}{\varepsilon \left(t\right)}=\frac{\sigma_{a}}{\varepsilon_{a}}e^{j\delta (\omega )\;}=\frac{\sigma_{a}}{\varepsilon_{a}}\cos\delta (\omega )+j\frac{{\;\sigma }_{a}}{\varepsilon_{a}}\sin\delta (\omega )=E^{\prime }\left(\omega \right)+j\;E^{\prime \prime }\left(\omega \right)=\hat{E}\left(\omega \right)$$

where $\sigma_{a}$ and $\varepsilon_{a}$ are the cyclic stress and strain amplitudes, respectively, and $E^{\prime }(\omega )$ and $E^{\prime \prime }(\omega )$ are the storage and loss moduli, respectively. $E^{\prime }$ represents the elastic energy stored during the cycle, while $E^{\prime \prime }$ represents the energy loss due to viscous dissipation caused by the viscoelastic material. Energy dissipation in polymer networks is caused by transitions in the material behaviors of polymers over various temperatures and frequency ranges.

#### 2.2.1. Relationship Between Relaxation Modulus and Complex Modulus

It is clear that the relaxation modulus in the time domain and the complex modulus in the frequency domain are related. If the sinusoidal input strain,$\;\varepsilon \left(t\right)=\varepsilon_{a}e^{j\omega t}$, is substituted into the hereditary integral in Equation (3), the time-dependent stress response can be expressed as follows [10]:

(7) $$\sigma \left(t\right)=\varepsilon_{a}j\omega \int_{-\infty }^{t}E\left(t-\tau \right)e^{j\omega \tau }d\tau$$

If a new dummy variable, s=t−τ, is defined, the above equation yields the following [10]:

(8) $$\sigma \left(t\right)=\varepsilon_{a}j\omega \int_{0}^{\infty }E\left(s\right)e^{j\omega \left(t-s\right)}ds\;\equiv \varepsilon_{a}e^{j\omega t}j\omega F\left[E(t)\right]\;\frac{\sigma \left(t\right)}{\varepsilon \left(t\right)}=\hat{E}\left(\omega \right)=j\omega F\left[E\left(t\right)\right]$$

The integral form in the above equation is the well-known Fourier transform of a function, which is the relaxation modulus.

The complex modulus at any temperature in the frequency domain can be obtained by substituting Equation (3) into Equation (7) [11]:

(9) $$\hat{E}\left(\omega ,T\right)=j\omega F\left[E\left(t,T\right)\right]=j\omega F\left[\sum_{i=1}^{N}E_{i}\exp\left(-\frac{t}{a_{T}\left(T\right)\tau_{i}}\right)\right]\;E^{\prime }\left(\omega \right)=E_{\infty }+\sum_{i=1}^{N}E_{i}\frac{\omega^{2}a_{\;T}^{2}\left(T\right)\tau_{i}^{2}}{1+\omega^{2}a_{T}^{2}\left(T\right)\tau_{i}^{2}};E^{\prime \prime }(\omega )=\sum_{i=1}^{N}E_{i}\;\frac{\omega a_{T}\left(T\right)\tau_{i}}{1+\omega^{2}a_{T}^{2}\left(T\right)\tau_{i}^{2}}$$

Similar to the reduced time, a reduced frequency can be defined as follows:

(10) $$\omega_{r}=a_{T}\left(T\right)\omega$$

Substituting Equation (9) into Equation (8) offers the master curves of the storage and loss moduli as follows:

(11) $$E^{\prime }\left(\omega_{r}(T)\right)=E_{\infty }+\sum_{i=1}^{N}E_{i}\frac{\omega_{r}^{2}\tau_{i}^{2}}{1+\omega_{r}^{2}\tau_{i}^{2}};E^{\prime \prime }(\omega )=\sum_{i=1}^{N}E_{i}\;\frac{\omega_{r}\tau_{i}}{1+\omega_{r}^{2}\tau_{i}^{2}}$$

**Figure 5:**
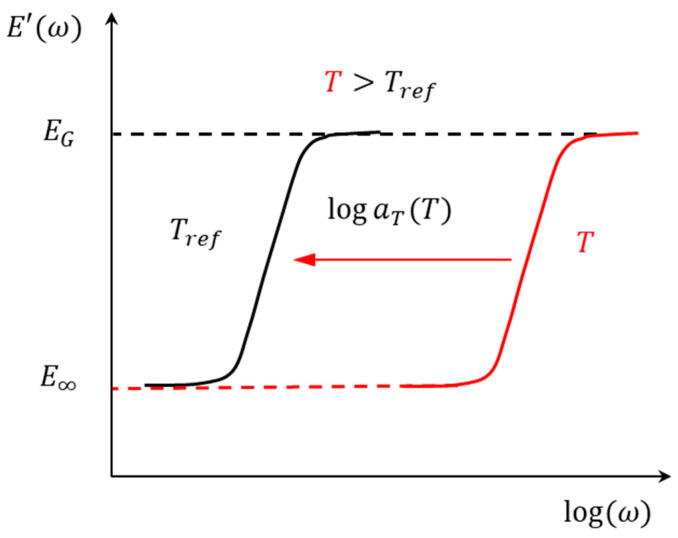
Illustration of material behavior of TRS polymers in frequency domain.

It is clear from Equation (10), that thermorheological simplicity is also applicable in the frequency domain. The effect of increasing temperature is simply a shifting of the viscoelastic response (plotted on a log frequency) to the right, without change in shape. This is illustrated schematically in Figure 5.

It is worth noting that the directions of shifting to determine the shift factors in the time and frequency domains are opposite. In the time domain, for $T>T_{ref}$, the master curve needs to be shifted to the left, while in the frequency domain, the master curve needs to be shifted to the right.

#### 2.2.2. Experimental Practice of TRS in the Frequency Domain

**Figure 6:**
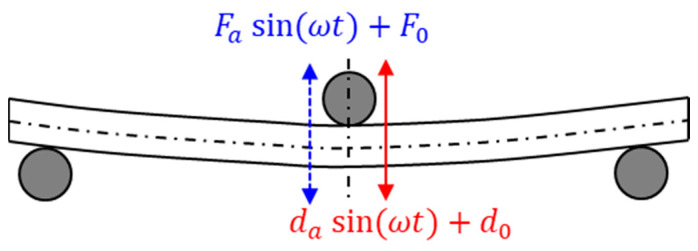
Three-point bend specimen and loading conditions.

A three-point bend test has been used for high-modulus polymers, while a simple tension fixture is used for low-modulus elastomers or thermoplastic films as they can take large elastic deformations. A three-point bending loading is illustrated schematically in Figure 6, where a preset harmonic displacement is applied at the center of the specimen. An initial preload, $F_{0}$, or displacement, $d_{0}$, is applied to maintain the continuous contact of the crosshead with the specimen. The measured oscillatory forces are used to obtain the storage modulus from the following governing equation [12]:

(12) $$E^{\prime }\left(\omega \right)=GF\cdot \frac{F_{a}\left(\omega \right)}{d_{a}\;\left(\omega \right)}\;GF=\frac{L^{3}}{48I}$$

where L is the length of the specimen, GF is a factor associated with the specimen geometry, I is the moment of inertia, and $F_{a}$ and $d_{a}$ are the oscillatory force amplitude and displacement amplitude, respectively. The loss modulus, $E^{\prime \prime }(\omega )$, can be obtained simply by multiplying the storage modulus with the tangent of the phase lag, tan⁡δ.

As mentioned earlier, the frequency- and time-dependent behaviors are interchangeable through Fourier transformation. Multi-frequency tests can be conducted at various temperatures to measure the viscoelastic behavior of a polymer.

## 3. Challenges Associated with Materials with Extreme Temperature Dependency

Multi-frequency tests are conducted at various temperatures to obtain the master curve in the frequency domain. The multi-frequency temperature (MFT) sweeping test is reviewed briefly here, and the challenges associated with EMCs are discussed.

### Extreme Change in Moduli over Testing Temperature

The most important test parameter for the three-point bend test is the oscillation displacement amplitude, which dictates the cyclic strain amplitude to be applied to the specimen. Typically, a single set of input parameters are used over the entire test. After measurement, an automated inbuilt TTS shifting algorithm is used to generate a master curve and the corresponding shift factors.

The elastic modulus of a highly filled EMC ranges from 20 to 30 GPa at room temperature. The modulus decreases rapidly to a sub-GPa range after passing its glass transition temperature. This extreme modulus change, which is uniquely seen in highly filled polymers, poses challenges to the routine practice of DMA measurements using a constant oscillation displacement amplitude. The following simple test was conducted to illustrate these challenges.

A commercially available EMC was used in the study. The base polymers of the EMC were epoxy resin with phenolic hardener, and it contained a very high filler content of 87% wt./wt. A bending specimen was fabricated using a custom-designed mold that had inner cavity dimensions of 50 mm × 6.9 mm × 3 mm. An EMC pellet was transferred into the cavity by applying a pressure of 7 MPa at a molding temperature of 130 °C. The EMC specimen was cured at 185 °C for 4 h after demolding.

A commercial DMA machine (TA DMA 850) was employed to conduct the three-point bending test using the following test parameters: (1) an oscillation amplitude of 10 μm, (2) a frequency range of 0.1 to 100 Hz (5 points/decade), and (3) a temperature range of 0 to 250 °C (10 °C/step). The frequency–temperature sweeping data were processed to produce a master curve and a set of shift factors by the TTS shifting program provided by the DMA manufacturer.

**Figure 7:**
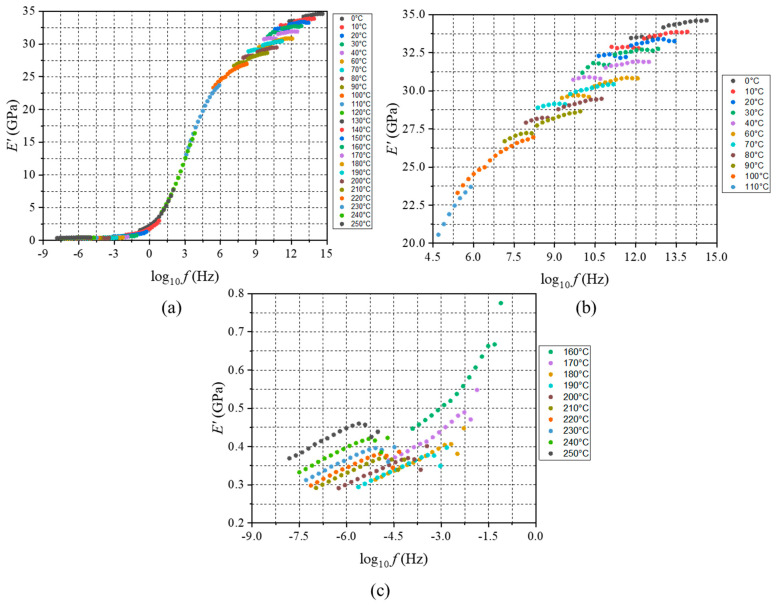
Horizontal shifting of EMC MFT data—(**a**) master curve generated after TTS shifting ($T_{ref}=130$ °C), (**b**) master curve in glassy region, and (**c**) master curve in rubbery region.

The master curve obtained through horizontal shifting is shown in Figure 7a. Discontinuities in the shifted data are evident. The glassy and rubbery regions are highlighted in Figure 7b,c, respectively. Although discontinuous, the data in the glassy region (0–110 °C) show a clear trend, while the data the rubbery region (170–250 °C) show no clear trend for temperature or frequency.

**Figure 8:**
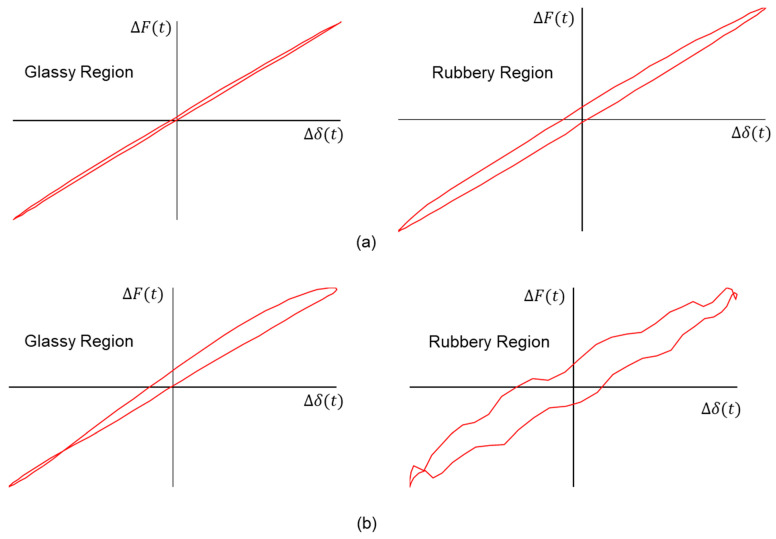
Representative oscillatory displacement and force data shown using Lissajous figures for (**a**) ideal responses and (**b**) unusual responses in glassy and rubbery regions, respectively.

The cause of these discontinuities can be explained further by examining the waveform of the oscillatory displacements and forces in the Lissajous figures. The plots are shown in Figure 8, where the representative responses of ideal behavior and unusual behavior are shown in (a) and (b), respectively. The force response should have a small but constant phase lag value across the entire cycle for the glassy region. However, the high-frequency response in the glassy region exhibits an asymmetric shape. The response in the rubbery region should be a smooth continuous Lissajous figure, but it contains a high level of random noise, implying that the machine may not be able to maintain proper contact with the specimen consistently throughout the test.

When a constant oscillation amplitude is used, the oscillatory stress amplitude changes significantly across the temperatures as the modulus decreases. For a typical EMC, the oscillatory stress amplitude at the lowest testing temperature can be 60 times as high as at that at the highest testing temperature, regardless of operating frequencies. Under these conditions, the machine’s behavior and response become significantly different across the test temperature range. An enhanced procedure to reduce these variations is warranted for testing EMCs.

## 4. Enhanced Procedure with Multiple Oscillatory Amplitudes and Monotonic Loading

An enhanced procedure is proposed to cope with the following problems with the current practice: (1) multiple oscillatory amplitudes and (2) monotonic loading tests for the glassy and equilibrium moduli. The procedure was implemented for a highly filled EMC.

### 4.1. Proposed Procedure

**Figure 9:**
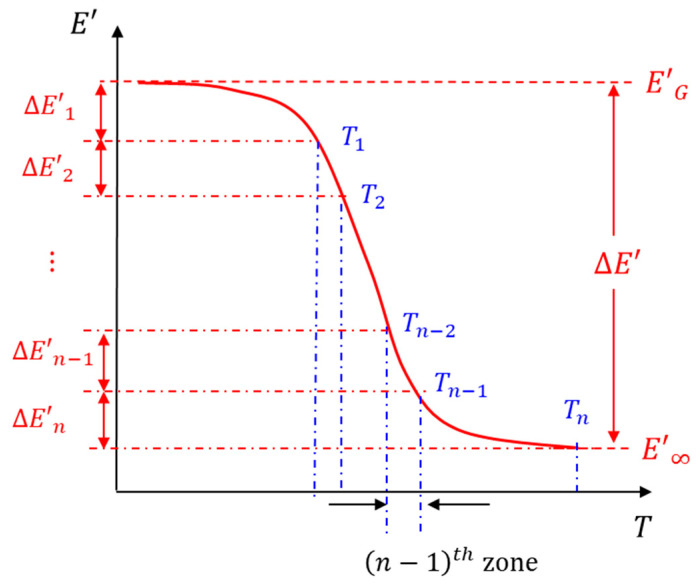
Illustration of 1 Hz temperature sweeping data.

DMA machines are routinely used to measure the glass transition temperature of polymers by conducting a temperature sweep at a 1 Hz oscillation frequency. A typical 1 Hz temperature sweep test is illustrated in Figure 9. The glassy modulus is denoted as $E_{G}$ and the equilibrium modulus is denoted as $E_{\infty }$. The glass transition region is defined as a region of the large change in storage moduli ($E^{\prime }$) over a short range of temperature. During this change, a polymer undergoes a physical transition from its glassy to rubbery states.

The proposed procedure uses different amplitudes across the temperatures during the MFT sweeping. The zones for the amplitudes are selected based on the storage modulus obtained from 1 Hz temperature sweeping data. This is illustrated schematically inFigure 9. The range of the storage modulus is divided equally, as follows:

(13) $$\Delta E_{i}=\frac{E_{G}-E_{\infty }\;}{n}$$

The corresponding temperatures are defined as $i^{th}$ zones, e.g., the ${\left(n-1\right)}^{th}$ zone covers a temperature range from $T_{n-2}$ to $T_{n-1}$. Then, the oscillation amplitude for each zone is selected to contain the variations in the cyclic stress amplitude of each zone in a small range.

**Figure 10:**
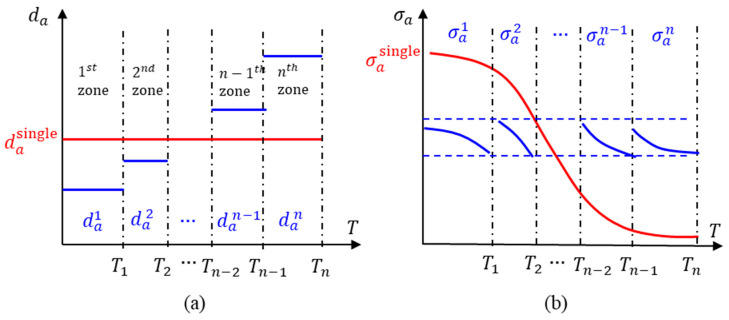
Proposed measurement procedure for MFT sweep, (**a**) shows the n levels of oscillation amplitudes for each zone, and (**b**) shows the expected variation in the cyclic stress amplitudes in each zone.

This concept is illustrated schematically in Figure 10, where (a) shows the n levels of oscillation amplitudes ($d_{a}^{1},\;d_{a}^{2}\ldots ,d_{a}^{n-1},\;d_{a}^{n}$) for each zone and (b) shows the expected variation in the cyclic stress amplitudes in each zone. The cyclic stress amplitude variation for a single oscillation amplitude (red line) is also shown in order to illustrate the reduction in cyclic stress amplitude variation.

The flexural stress amplitude, $\sigma_{a}$, in the beam under three-point bending test conditions can be determined using the following equation [12]:

(14) $$\sigma_{a}=\frac{{6E}^{\prime }td_{a}}{L^{2}}$$

where $E^{\prime }$ is the storage modulus; $d_{a}$ is the oscillation amplitude; and t and L are the thickness and length of the beam, respectively. This equation can be used to determine the oscillation amplitudes of N−zones such that the variations in the cyclic stress amplitude can be reduced. These criteria can be expressed using the average cyclic stress amplitude for each zone, as follows:

(15) $${\left[\sigma_{a}^{1}\right]}_{avg}\approx {\left[\sigma_{a}^{2}\right]}_{avg}\approx \ldots \approx {\left[\sigma_{a}^{n-1}\right]}_{avg}\approx {\left[\sigma_{a}^{n}\right]}_{avg}\;\;{{\left[E^{\prime }\right]}^{1}}_{avg}d_{a}^{1}\approx {{\left[E^{\prime }\right]}^{2}}_{avg}d_{a}^{2}\;\approx \ldots \approx {{\left[E^{\prime }\right]}^{n-1}}_{avg}d_{a}^{n-1}\approx \;{{\left[E^{\prime }\right]}^{n}}_{avg}d_{a}^{n}$$

The ends of the master curve, i.e., the glassy and equilibrium moduli, are always prone to having more uncertainties during shifting. At these extreme conditions, the EMC behaves elastically, and monotonic loading is more ideal for these properties than cyclic loading. In this second step, the same setup and specimen are used to conduct tests under monotonic loading. The temperatures for the monotonic load testing should be the lowest temperature and the highest temperature of testing. The first monotonic testing at the lowest temperature will determine the glassy modulus, while the second testing at the highest temperature will determine the equilibrium modulus. These values will be used to guide the procedure to construct the master curve.

### 4.2. Implementation

**Figure 11:**
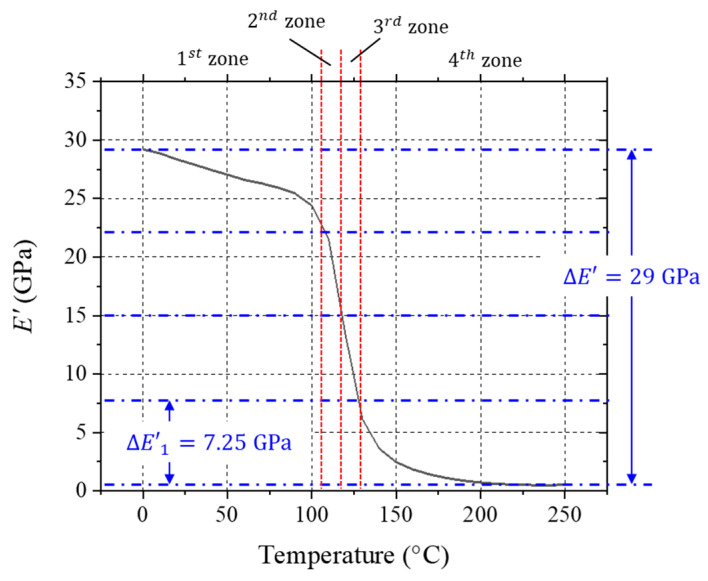
The 1 Hz temperature sweeping data of the EMC.

The 1 Hz testing results of the EMC specimen are shown in Figure 11. The modulus at 0 °C (glassy) and 250 °C (equilibrium) were 29.5 GPa and 0.5 GPa, respectively. The DMA machine had been practiced successfully for unfilled or lightly filled thermosets using a single amplitude during MFT sweeping. The changes in storage modulus over the glass transition region of unfilled or lightly filled thermosets have been reported to vary from 5 to 8 GPa [3,13]. Using these variations as a guideline, the storage modulus was divided into four zones of 7.25 GPa in this study.

Temperature ranges for each zone were chosen based on the following divisions: 1st zone (0 to 105 °C), 2nd zone (105 to 115 °C), 3rd zone (115 to 135 °C) and 4th zone (135 to 240 °C). The average storage modulus of the four zones were determined to be 27 GPa, 21.5 GPa, 16.4 GPa, and 2.1 GPa, respectively. Considering the specification for TA DMA 850, the amplitude for each zone was determined by Equation (13) using the average values of storage modulus; they were 4μm, 5μm, 7 μm, and 52 μm for the four zones. Under these conditions, the average stress amplitude was expected to be approximately 0.77 MPa in all four regions. This stress level was sufficiently low to not affect the repeated measurements of an MFT sweeping test.

**Figure 12:**
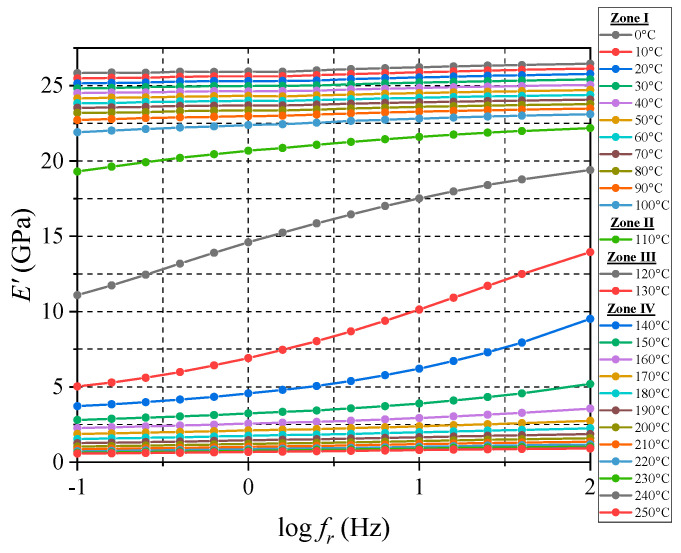
Frequency data obtained from MFT sweep test with multiple strain amplitude levels.

An MFT sweeping test was conducted using the four oscillation amplitudes, a temperature range of 0 to 250 °C and a frequency range of 0.1 to 100 Hz. The results are shown in Figure 12, Clear trends in storage moduli are evident in all four zones.

**Figure 13:**
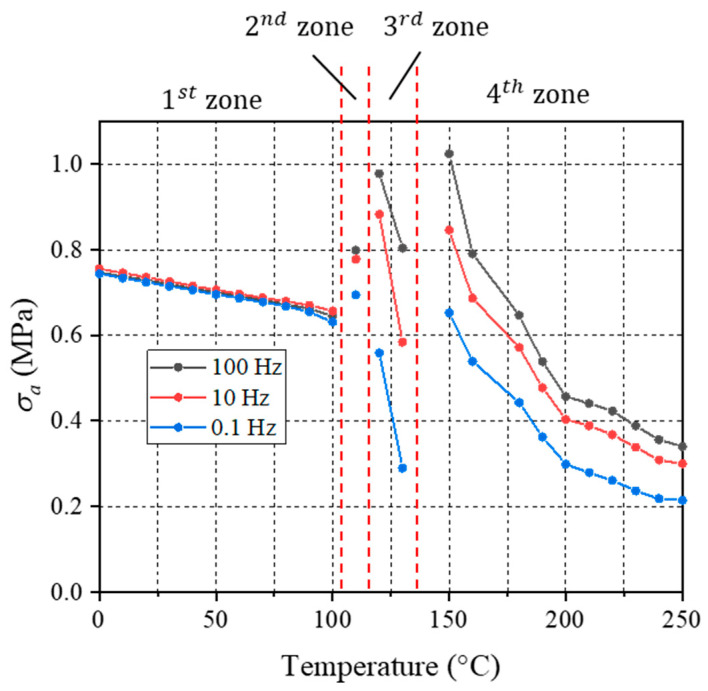
Oscillatory stress amplitudes of proposed procedure.

Figure 13 shows a plot of oscillatory stress amplitude vs. temperature at three different frequencies: 0.1 Hz, 10 Hz, and 100 Hz. The maximum stress ratio ($\sigma_{max}/\sigma_{min}$) was reduced from 50, for a single-amplitude MFT sweep, to 5 after the implementation of the proposed procedure.

**Figure 14:**
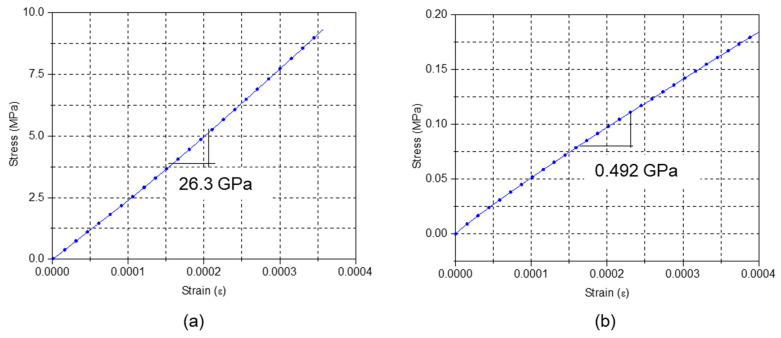
Monotonic loading test—(**a**) 0 °C and (**b**) 250 °C.

**Figure 15:**
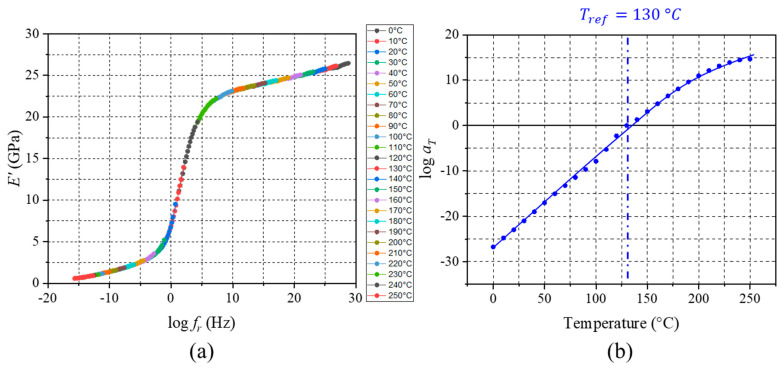
(**a**) $E^{\prime }$ master curve and (**b**) shift factors of EMC obtained by proposed procedure.

As the second step, monotonic load testing was conducted on the same EMC specimen. The stress vs. strain plots obtained from the monotonic tests at 0 °C and 250 °C are shown in Figure 14. The glassy and equilibrium moduli are 26.3 GPa and 0.492 GPa, respectively. It is worth noting that the data show an ideal linear relationship, which confirms that the EMC behaved elastically at those two extreme temperatures. TTS shifting was followed to generate a master curve and a set of shift factors. The master curve and shift factors are shown in Figure 15a,b, respectively.

Both a reduction in the cyclic stress amplitude variation and the well-characterized glassy and equilibrium moduli can be attributed to the easy construction of the storage modulus master curve shown in Figure 15a. The smooth and continuous modulus master curve offers some guidelines for the enhanced accuracy of the proposed procedure.

### 4.3. Determination of Generalized Maxwell Model Constants

The Prony series can be determined from the storage master curve. In the proposed procedure, a set of relaxation time constants, $\tau_{i}$, are first assumed, typically equally spaced in the log time scale, and then the generalized Maxwell model constants, $E_{i}$, are calculated using Equation (10) by non-linear regression.

It is worth noting that the range of decades used in the master curve is readily assessed by the shift factors at the temperature extremes. For the EMC tested in the study, the shift factors were $\log a_{T}=-26.76$ and 14.66 at 0 °C and 250 °C, respectively. Considering this decade range, the total number of the relaxation time constants should be 40, and thus the values $\tau_{i}$ range from $10^{-26}$ to $10^{14}$.

**Figure 16:**
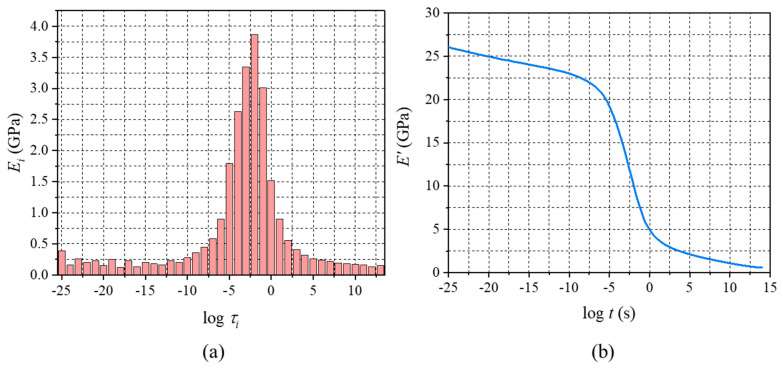
(**a**) Generalized Maxwell model constants ($E_{i}$) vs. $\log\tau_{i}$, and (**b**) relaxation modulus master curve.

A MATLAB program to determine the generalized Maxwell model constants from the storage modulus master curves (Figure 14) is presented in Appendix A. The generalized Maxwell model constants, $E_{i}$, along with the relaxation time constants, $\tau_{i}$, are shown graphically in Figure 16a. 

The generalized Maxwell model constants and the shift factors could be used to produce the relaxation modulus master curve using Equation (1). The results are shown in Figure 16b. The MATLAB code to produce the relaxation master curve is also shown in Appendix A.

## 5. Discussions

### 5.1. Virtual Testing Using the Relaxation Modulus

A numerical analysis was conducted to simulate the DMA testing to ensure the accuracy of generalized Maxwell model constants. The same geometry of 50 mm × 6.9 mm × 3 mm using the simply supported ends was used in the simulation. The cyclic strain amplitudes used in the actual DMA testing were applied at the center of the specimen. The cyclic responses were obtained by the harmonic analysis module in ANSYS Workbench 2023 R2.

**Figure 17:**
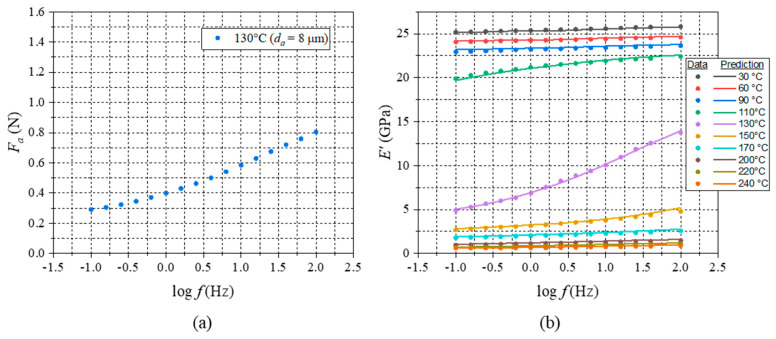
(**a**) $F_{a}$ vs. log⁡f at 130 °C and (**b**) comparison of experimental storage modulus data with prediction from virtual DMA experiment.

The output of the numerical analysis consisted of the real and imaginary components (amplitude) of the oscillatory force, $F_{a}$, over a cycle. To illustrate the output, the real component of the oscillatory force at 130 °C was plotted as a function of the applied frequencies in Figure 17a. From the numerical results, the storage moduli at the test frequencies could be readily calculated using Equation (12).

A virtual DMA test [14] was conducted at various temperatures in the glassy region, the glass transition region, and the rubbery region of the EMC. The predictions from the virtual DMA were compared with the actual experimental data (in Figure 12) which are shown in Figure 17b. The results (overall $R^{2}=0.99$) clearly indicated that the generalized Maxwell constants (i.e., Prony series) determined from the proposed DMA test procedure accurately described the EMC’s viscoelastic behavior in both the time and frequency domains.

### 5.2. Master Curve Calculation with Horizontal and Vertical Shifting

**Figure 18:**
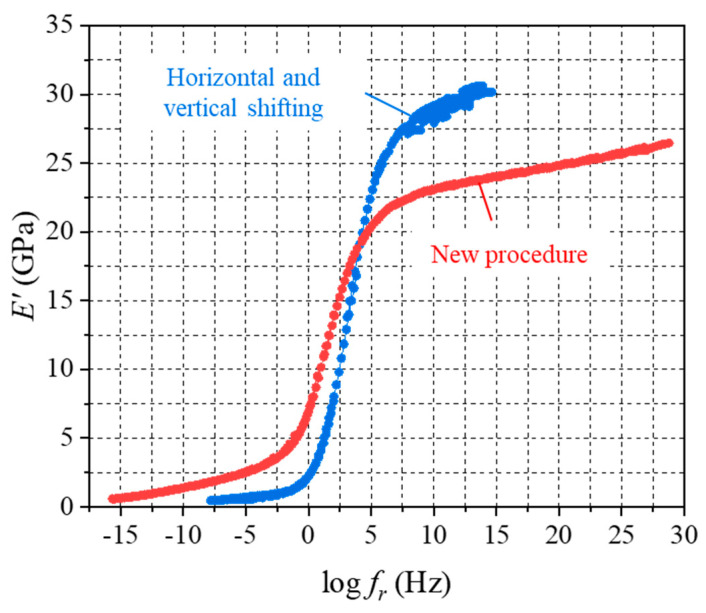
A comparison of the master curve generated using the horizontal and vertical shifting technique with the new master curve.

Some inbuilt shifting procedures of the advanced commercially available DMA machines offer an automatic shifting procedure in both the horizontal and vertical directions to maximize the overlaps of the MFT data in the master curve. The data shown in Figure 7 (blue) were reprocessed using the shifting options in both directions. The results are shown in Figure 18.

More ideal overlaps in the glassy region and rubbery regions are observed, compared with the data in Figure 7. Two critical technical issues associated with this procedure need to be addressed for proper practice. First, it has been reported that some rare polymers exhibit a vertical shifting behavior (i.e., shifting along the modulus direction). To the best of the authors’ knowledge, none of the advanced thermosets used in semiconductor packaging technologies have demonstrated this behavior.

More importantly, the commercial numerical tools for LVE analyses do not account for this behavior. In fact, this behavior is not an actual material response but an experimental artifact that must be corrected. The storage modulus master curve obtained from the proposed procedure is also shown in Figure 18 for comparison. The glassy modulus is overestimated by 30% and the equilibrium modulus is underestimated by 10% when the two-direction shifting procedure is employed. Additionally, the decades of the master curve obtained by the two-direction shifting procedure are significantly smaller than the actual behavior, which results in the significant underestimation of relaxation times in viscoelastic analysis.

## 6. Conclusions

An EMC was tested by a commercial DMA machine using the automated test procedure, i.e., by applying a single amplitude over all the testing temperatures. The master curve showed discontinuities in the glassy region while no clear trend was visible in the rubbery region. These were caused by the extremely large change in the EMC modulus over the testing temperatures, which was attributed to a very large amount of filler contents over 80%.

An advanced procedure was proposed to cope with the problem. The advanced procedure contained two parts: (1) the use of different oscillation amplitudes across various zones during MFT sweeping to reduce the cyclic stress amplitude variations, and (2) the use of monotonic testing to measure the glassy and equilibrium modulus in the time domain. The proposed procedure was implemented for a highly filled EMC. The results showed a clear trend in the storage modulus across all four zones. With the aid of the glassy modulus and equilibrium modulus, which were measured separately using monotonic loading, a master curve was accurately constructed.

The generalized Maxwell model constants were determined from the storage modulus master curve. The accuracy of the model constants was evaluated by conducting a virtual DMA test using a harmonic analysis module in the ANSYS program. The results clearly indicated that the generalized Maxwell constants (i.e., Prony series) determined from the proposed DMA test procedure accurately described the EMC’s viscoelastic behavior in both the time and frequency domains.

## Data Availability

The data will be made available upon request.

## List of Abbreviations

DMA	dynamic mechanical analysis
EMC	epoxy-based molding compound
TTS	time–temperature superposition
FO-WLP	fan-out wafer-level packaging
SEM	scanning electron microscope
LVE	linear viscoelasticity
TRS	thermorheological simplicity
MFT	multi-frequency temperature

## Appendix A. MATLAB Codes

I. The MATLAB code used for fitting the $E^{\prime }$ master curve

This section shows the MATLAB code used to determine the Prony constants, $E_{i}$, and $E_{\infty }$ using known $\tau_{i}$ values (chosen at decade intervals) from the E′ master curve.

%% Reading data file

A=xlsread("Freq_mastercurve.xlsx");

[m,n]=size(A);

MS=[];

p=log10(2*pi);

for i=1:2:n−1

  temp=[p+A(:,i),A(:,i+1)];

  MS=[MS;temp];

end

%Select the range of the time-constant powers for fitting

lowest_power = −floor(max(MS(:,1)));

highest_power = −ceil(min(MS(:,1)));

%% Choosing number of terms-default is (max power + min power + 1)

%Select number of terms (User Input needed)

%Default is each term assigned for one decade)

number_of_terms = ceil((abs(lowest_power)+abs(highest_power)+1));

MS(:,1)=10.^(MS(:,1));

MS(:,2)=MS(:,2);

%% Setting up time constant matrix

%The power of 10^50 has been additionally added to simulate E_infity and

%for ease of matrix multiplication

global tau

tau=logspace(highest_power,lowest_power,number_of_terms);

tau=[10^(highest_power+50) tau];

%% Least Square Optimization Program-By default uses gradient descent

E_initial=ones(number_of_terms+1,1);

E_lb=zeros(number_of_terms+1,1);

E_ub=20*ones(number_of_terms+1,1);

[E,resnorm,residual,exitflag,output,lambda,jacobian]=lsqcurvefit(@pronyseries,E_initial,MS(:,1),MS(:,2),E_lb,E_ub);

%% Post-processing

%Output

val=(MS(:,1).^2*tau.^2)./(1+MS(:,1).^2*tau.^2);

y_out=val*E;

%R^2 calculation

mn=mean(MS(:,2));

SStot=sum((MS(:,2)-mn).^2);

SSres=sum((MS(:,2)-y_out).^2);

Rsq=1−SSres/SStot;

%Plot for comparion

figure(1);

%bar(E);

%hold on

figure(1);

semilogx(MS(:,1),MS(:,2));

hold on

semilogx(MS(:,1),y_out);

%Output file

logwout=[log10(MS(:,1)), MS(:,2), y_out];

%% Plotting Mastercurve in Time Domain

time=logspace(lowest_power-1,highest_power+1,1000);

time=time’;

X=time./tau.^1;

X=exp(−X);

E_timedomain=X*E;

%semilogx(time,E_timedomain);

out_timedomain=[time,E_timedomain];

prony_out=[tau’,E];

%% Function for evaluating Prony Series-Uses matrix algebra only for efficiency

function y_fit=pronyseries(E,x_data)

  global tau

  val=(x_data.^2*tau.^2)./(1+x_data.^2*tau.^2);

  y_fit=val*E;

end

II. The MATLAB code used to plot the master curve in the time domain

This section shows the MATLAB code used to plot the master curve in the time domain using the Prony constants obtained from the above program.

% Select the range of time for plot

time=logspace(−20,10,100);

%Select shift factors to plot for, i.e., Temperature

aT=[0];

n=size(aT);

E_out=[];

for i=1:1

  t=10^(aT(i))*tau;

  X=exp(−time’./t.^1);

  y=X*E;

  time_acc=10^(−aT(i))*time;

  E_out=[E_out, time’, y];

end

%Semilogx plot of the master curves.

semilogx(E_out(:,1),E_out(:,2));
